# Timing and Predictive Value of Clinical Conditions Preceding Multiple Sclerosis in the UK Biobank

**DOI:** 10.1002/acn3.70119

**Published:** 2025-06-26

**Authors:** Andrea Nova, Teresa Fazia, Giovanni Di Caprio, Davide Gentilini, Luisa Bernardinelli, Roberto Bergamaschi

**Affiliations:** ^1^ Department of Brain and Behavioral Sciences University of Pavia Pavia Italy; ^2^ Bioinformatics and Statistical Genomic Unit IRCCS Istituto Auxologico Italiano Milan Italy; ^3^ Multiple Sclerosis Research Center IRCCS Mondino Foundation Pavia Italy

**Keywords:** comorbidities, multiple sclerosis, prediction, prodrome

## Abstract

**Objectives:**

Multiple sclerosis (MS) patients often experience a higher incidence of clinical conditions before diagnosis, suggesting a prodromal phase. However, their predictive value and temporal trajectories remain underexplored. We investigated these aspects using the large UK Biobank's population‐based cohort, which provided clinical history through ICD‐10 diagnosis codes.

**Methods:**

We assessed associations between 600 clinical conditions and MS risk in 477,421 individuals using Cox models adjusted for demographics, smoking, and MS polygenic risk score (MS‐PRS). Statistically significant conditions were included in a LASSO Cox regression (five‐fold cross‐validation on 70% training set) to identify key predictors, with performance assessed by the C‐index and age‐dependent area under the curve (AUC) in the 30% test set. Lastly, temporal trajectories of MS‐associated conditions were analyzed in MS cases.

**Results:**

We identified 192 conditions associated with MS, of which only ~20% were onset symptoms. Integrating these conditions into a predictive model already including demographics and smoking, improved the C‐index from 0.65 to 0.71. Among the 30 model‐selected best predictors, ~25% were prodromal conditions, including neuromuscular diseases, thromboembolism, and depression which typically occurred more than five years before MS diagnosis. Including MS‐PRS further increased the C‐index to 0.78, with an age‐dependent AUC exceeding 0.80 in individuals over 50 years. Trajectory analysis highlighted migraine as a common early diagnosis, often followed by hypertension, depression, and dorsalgia.

**Interpretation:**

Our findings highlight early conditions and diagnostic trajectories of MS, supporting the existence of a prodromal phase. These insights could improve MS prediction and facilitate earlier detection, particularly for late‐onset cases.

## 1 Introduction

Multiple sclerosis (MS) is a complex chronic inflammatory and demyelinating disease affecting the central nervous system (CNS) characterized by varying clinical presentations and significant heterogeneity in disease progression [1]. While MS has traditionally been considered a disease of sudden onset, increasing evidence supports the existence of an MS prodromal phase during which subtle, non‐specific symptoms and health conditions may emerge before the disease meets diagnostic criteria [2, 3]. Several studies have shown that individuals who later develop MS often exhibit increased healthcare utilization and a higher incidence of various clinical conditions years before their diagnosis [4, 5, 6, 7, 8, 9]. These early conditions include not only physical symptoms such as pain, migraine, urinary tract issues, sensory disturbances, and fatigue, but also cognitive problems and neuropsychiatric conditions like anxiety and depression [10, 11, 12, 13, 14, 15, 16, 17]. Moreover, increased levels of serum neurofilament light chain, a biomarker of neuronal damage, have been observed even six years before the clinical MS onset [18]. Together, these findings suggest a detectable prodrome, analogous to that seen in other neurodegenerative diseases [2].

However, while several studies have established associations between pre‐diagnostic conditions and MS, few have assessed their overall predictive value or examined how these conditions evolve over time. Moreover, many existing studies have employed nested case–control designs based on fixed time windows (e.g., 5 or 10 years before MS diagnosis), which may overlook earlier predictive conditions and potentially lead to biased results by emphasizing conditions closer to the MS diagnosis [19].

To address these limitations, we used data from the large UK Biobank's population‐based cohort, which includes ICD‐10 diagnoses codes, along with their dates, recorded throughout individuals’ lifetime. This data allowed us to implement a time‐to‐event framework which more accurately accounts for the timing of the individuals’ diagnostic history prior to the MS diagnosis without fixing any arbitrary time window.

Specifically, we aimed to (i) identify clinical conditions significantly associated with an increased risk of being diagnosed with MS, characterizing their relationship with MS (e.g., onset symptoms, prodromes) and assessing their timing in relation to MS diagnosis, (ii) evaluate their predictive value, and (iii) identify potential temporal diseases trajectories leading to MS. Moreover, we evaluated the improvement in predictive accuracy by first considering conditions typically occurring many years prior to MS, providing potential insights into the MS prodromal phase.

Such analyses could provide insights into the progression of these clinical conditions to an MS diagnosis, enhancing the characterization of the prodrome phase and helping to identify early predictive clinical signs. These objectives are particularly relevant due to the recently highlighted increase in late‐onset MS (LOMS) cases, which may result from the accumulation of several health conditions with age [20, 21, 22].

## 2 Methods

This study is an observational study conducted according to the Strengthening the Reporting of Observational Studies in Epidemiology (STROBE) reporting guidelines for observational studies.

### 2.1 Institutional Review Board Approval

This work was performed using data from UK Biobank (REC approval 11/NW/0382). All participants gave informed consent on Biobank registration and are free to withdraw from the study at any point, at which point their data are censored and cannot be included in further analyses.

### 2.2 Data

The UK Biobank [23] offers a comprehensive range of health‐related phenotypes and genetic data collected from individuals recruited between 2006 and 2010 and aged 40 to 70.

### 2.3 Clinical History and Multiple Sclerosis Diagnosis

At the time of recruitment in the UK Biobank study, participants' clinical histories were linked from several sources: (i) hospital admissions, (ii) primary care records, (iii) death registries, and, when applicable, (iv) self‐reports (based on previous diagnoses made by a physician and reported by the individuals at the time of recruitment through a questionnaire). Diagnoses made after study recruitment were continuously recorded throughout the follow‐up period.

The diagnosis codes were based on the 10th revision of the International Classification of Diseases (ICD‐10). In our analysis, we initially considered a total of 1785 ICD‐10 codes. First, 119 ICD‐10 codes concerning childbirth and congenital conditions were excluded (i.e., chapters O, P, and Q). Subsequently, when deemed appropriate, we aggregated closely related conditions in 599 subgroups to reduce redundant information and avoid multicollinearity issues in the statistical analyses. Lastly, we further considered pregnancy as a clinically important event and retrieved the information using self‐reported questions related to the number of live births and the age at first live birth. A total of 600 clinical conditions, as reported in Table S1, were then included in the analysis. This filtering process flow is depicted in Figure S1.

MS diagnosis, representing our outcome, was defined according to the ICD‐10 diagnosis code G35. Age at diagnosis for each clinical condition was determined by subtracting the date of birth from the diagnosis date.

### 2.4 Covariates

In our analysis, we considered several confounders, i.e., known MS risk factors, for the relationship between clinical conditions and MS diagnosis. These were collected at the initial assessment visit and included sex, year of birth, ethnicity (i.e., White, Non‐White), place of birth (i.e., England, Wales, Scotland, Ireland/North Ireland, Europe, Asia/Oceania, Africa, America), smoking, and a Multiple Sclerosis Polygenic Risk Score (MS‐PRS) provided by the UK Biobank. Other established MS risk factors that manifest as clinical conditions (e.g., infectious mononucleosis) were already captured within the set of clinical conditions analyzed in our study. Individuals with missing data on ethnicity, place of birth, or MS‐PRS were excluded from the analysis (*N* = 24,933). Details on confounders definition and derivation from UK Biobank data fields are described in Table S2.

### 2.5 Statistical Analysis

Descriptive statistics were calculated for all the analyzed variables, and differences were examined between individuals diagnosed with MS and those unaffected. Missing data for age at smoking initiation and age at first live birth (< 1%) were imputed using multiple imputation [24]. Statistical analyses were performed using RStudio 2024.12.1, and an R code template was included in the Supporting Information.

### 2.6 Associations Between Clinical Conditions and Multiple Sclerosis

**FIGURE 1:**
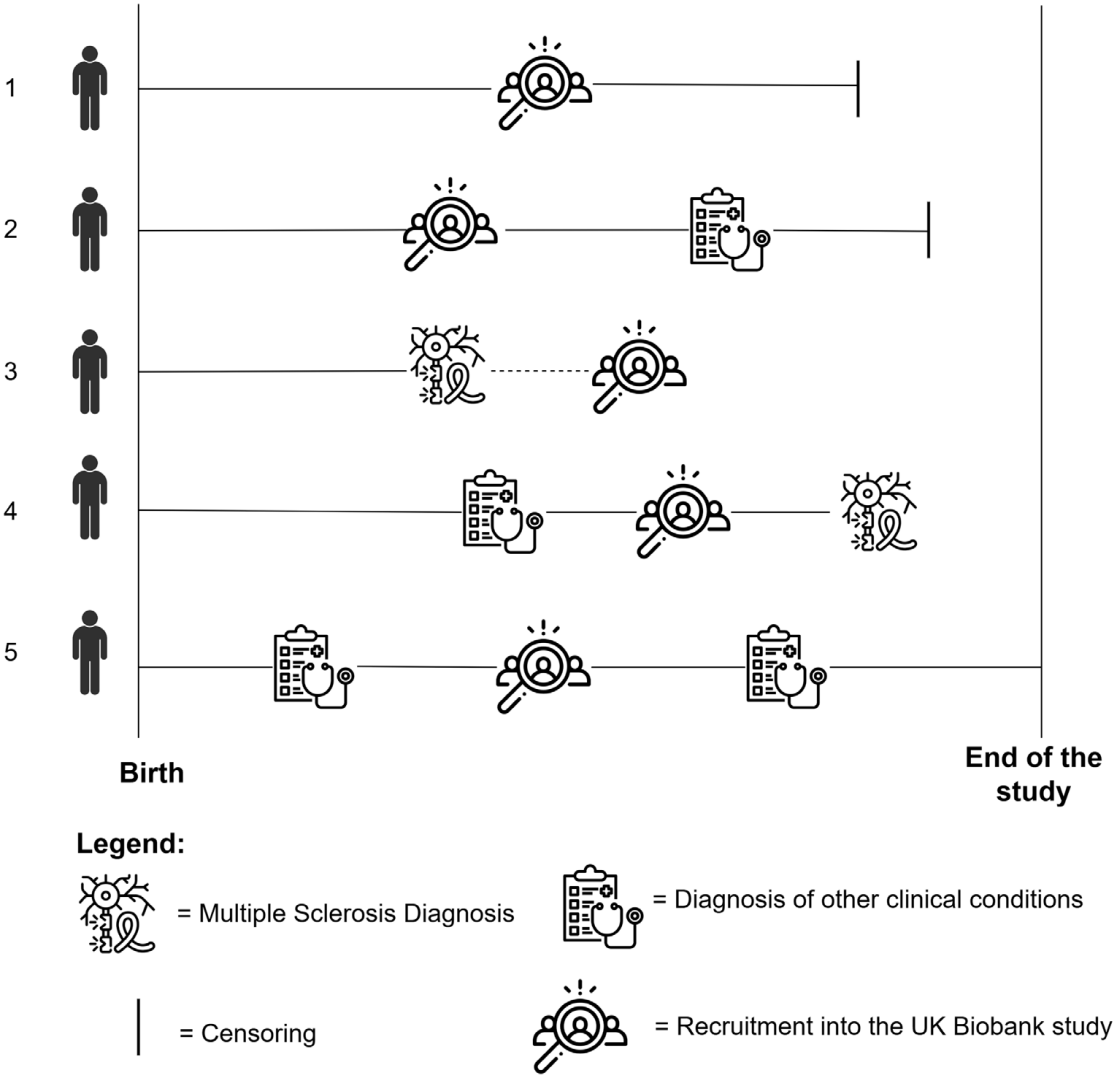
Observation periods, illustrated as continuous lines in our time‐to‐event analysis, are shown in five examples. For all individuals, observation time begins at birth. Individual 1 was never diagnosed with multiple sclerosis (MS) or any other clinical condition and was censored due to death or loss to follow‐up. Individual 2 was not diagnosed with MS but developed another clinical condition after recruitment into the UK Biobank (UKBB), before being censored. Individual 3 was diagnosed with MS prior to UKBB recruitment and did not develop any other clinical conditions; the dashed line represents the period between MS diagnosis and UKBB recruitment, which is not included in the observation time. Individual 4 had a clinical condition diagnosed before UKBB recruitment and was later diagnosed with MS during follow‐up. Individual 5 was not diagnosed with MS but developed two distinct clinical conditions, one before and one after UKBB recruitment, and was censored at the end of the study (December 31, 2022).

We examined whether specific clinical conditions were associated with MS diagnosis using a time‐to‐event analysis. Given the availability of the participants' diagnoses recorded both prior and after their recruitment in the study, we conducted our analysis based on a retrospective cohort study design. Specifically, we considered the observation time as starting from birth and continuing up to the earliest date among MS diagnosis, death, loss to follow‐up, or the study's end (December 31, 2022). An illustration of observation times is depicted in Figure 1. Each condition was treated as a time‐varying variable, switching from “No” to “Yes” at the date of diagnosis, and we estimated their associations with MS diagnosis using Cox proportional hazards models adjusted for sex, year of birth, ethnicity, place of birth, MS‐PRS, and smoking. To account for multiple testing, we applied a false discovery rate (FDR) correction at 0.05, and conditions significantly associated with MS diagnoses were classified as “MS‐associated conditions.” To understand when these conditions typically occur before MS diagnosis, we calculated the median time between the condition and MS diagnoses. Conditions were classified based on their timing of appearance in relation to MS diagnosis, i.e., > 5 years, 3–5 years, 1–3 years, or within 1 year, as well as based on their clinical relationship with MS, i.e., onset symptoms, prodromal conditions, risk/protective factors, or unknown relationship.

### 2.7 Evaluating the Predictive Accuracy of MS‐Associated Conditions

To determine which conditions best predict MS diagnosis, we split our dataset into a training/validation set (70%) and a test set (30%). We first built a baseline model using demographics, i.e., sex, year of birth, ethnicity, place of birth, and smoking, and used a LASSO Cox regression with five‐fold cross‐validation (CV) in the training set for parameter optimization. We then assessed the model's accuracy in the test set through Harrell's C‐index [25]. C‐index estimates the probability that an individual diagnosed with MS has a higher predicted MS risk than one diagnosed later. We then tested whether including MS‐associated conditions improved predictions accuracy. Conditions were first added in stages based on how long before MS diagnosis they tend to occur, i.e., a median time > 5 years, > 3 years, and > 1 year, respectively, and subsequently all conditions were added among the predictors. To rank the most important conditions based on their predictive value, we used permutations [26]. Furthermore, we calculated the time‐dependent Incident Cases/Dynamic Controls Area Under the Curve (AUC^I/D^(t)) to evaluate predictive accuracy as a function of age. This measure represents the probability that an individual diagnosed with MS at age t has a higher predicted MS risk score than an individual of the same age who is MS‐free at that time [27]. Finally, we repeated the process by adding the MS‐PRS among the predictors to evaluate whether clinical history improved prediction beyond genetic risk. Further details can be found in the Supporting Information.

### 2.8 Clinical Conditions Trajectories Leading to Multiple Sclerosis Diagnosis

Lastly, we investigated temporal disease trajectories leading to an MS diagnosis, using the method proposed in [28]. First, we retrospectively examined the sequence of clinical conditions in individuals diagnosed with MS. Second, we assessed all possible ordered pairs of MS‐associated conditions, denoting them as Condition 1 (C1) and Condition 2 (C2). Only conditions occurring in at least 25 MS cases (~1%) and conditions' pairs present in at least 10 individuals were considered. Fisher's exact tests were used to determine whether C2 was diagnosed after C1 in more than 50% of individuals with both conditions. Significant associations were identified using an FDR threshold of 0.05. Third, we conducted a nested case–control study to confirm the strength of these associations. Individuals diagnosed with C2 were matched to individuals without C2 at the same age, and a conditional logistic regression model was used to estimate the odds ratio for C1 → C2, adjusting for all confounders listed above. C1 → C2 pairs significantly associated with an FDR < 0.05 were then merged into broader trajectories. For example, if C1 → C2 and C2 → C3 were both significant, they were combined into a single trajectory (C1 → C2 → C3). These trajectories were visualized using Cytoscape Desktop v3.10.2 to better understand the temporal sequence of conditions leading to an MS diagnosis.

## 3 Results

We analyzed data from 477,421 individuals, mostly born in England between 1934 and 1971, and of predominantly white ethnicity. Individuals had an average observation time of 72 years (SD: 8.3), during which 2463 had been diagnosed with MS. Nine MS diagnoses (4%) occurred between 15 to 17 years old, i.e., pediatric‐onset MS (POMS), at a mean age of 16.8 years (SD: 1.0), 1598 (65%) between 18 to 49 years old, i.e., adult‐onset MS (AOMS), at a mean age of 37.9 years (SD: 7.9), and 856 (35%) from the age of 50, i.e., LOMS, at a mean age of 59.9 years (SD: 7.9). The discrepancy between the mean age at AOMS and the known average AOMS onset (around 33 years) mainly derives from the cohort's study design, other than from the improvement in the MS diagnostic criteria over the years. As the cohort aged, the absence of younger individuals born after 1971 limited the inclusion of newly diagnosed younger cases as it is reflected by the progressive increase in the average age at MS diagnosis across calendar years: 27.2 (SD: 5.5) in 1980, 31.8 (SD: 7.4) in 1990, 37.3 (SD: 9.1) in 2000, and 45.5 (SD: 13.2) in 2022 (end of the study). Demographic characteristics of our sample are reported in Table S3, comparing individuals with and without an MS diagnosis.

### 3.1 Associations Between Clinical Conditions and Multiple Sclerosis Diagnosis

**FIGURE 2:**
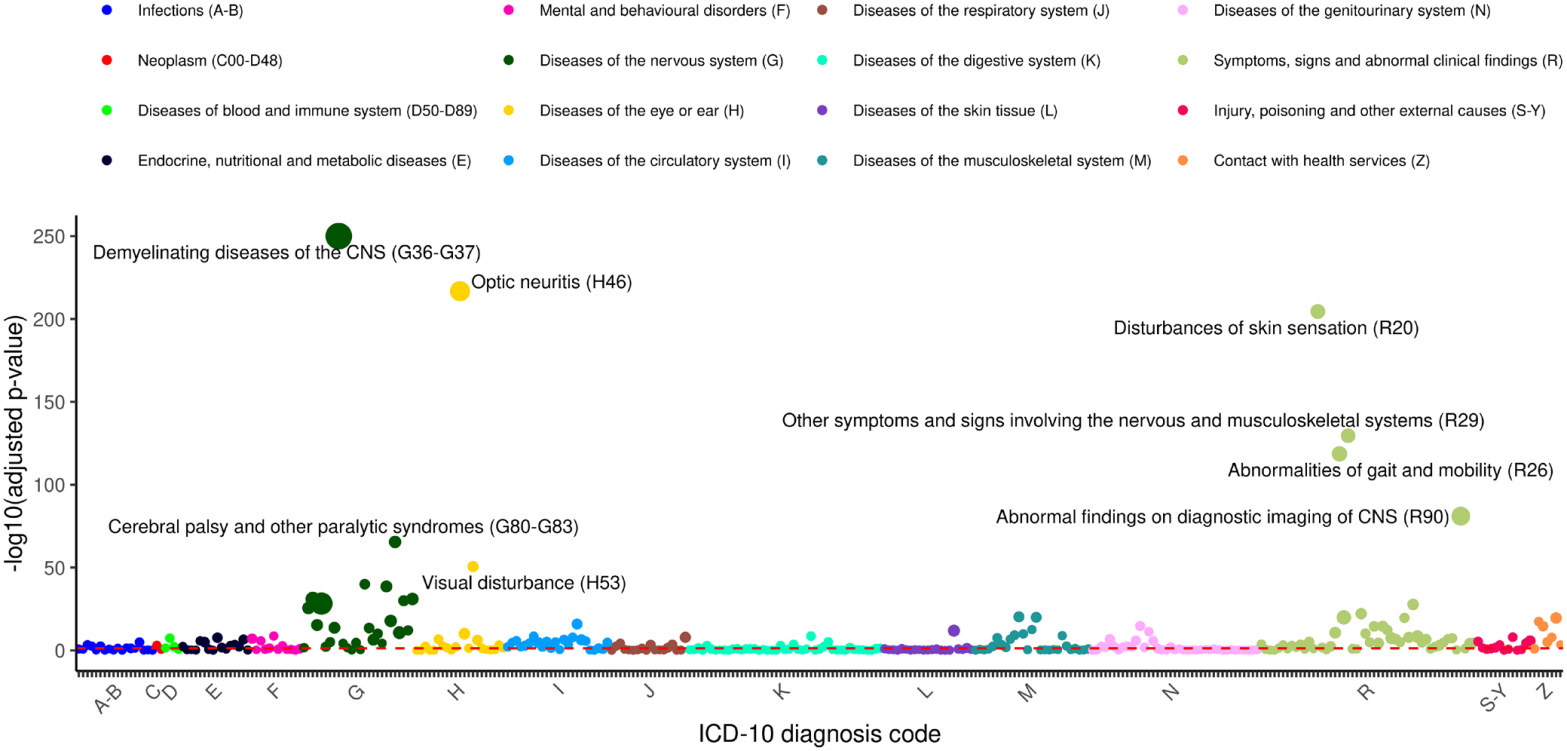
Univariate associations between clinical conditions estimated through a Cox proportional hazard model adjusted for demographics, smoking, and genetic risk of Multiple Sclerosis, grouped by different ICD‐10 code chapters, and Multiple Sclerosis diagnosis. *Y*‐axis denotes the −log_10_(adjusted *p*‐value), where the adjusted *p*‐value was obtained through the Benjamini–Hochberg correction, while the size of the dots represents the magnitude of the Hazard Ratio (the higher the Hazard Ratio the larger the dot).

The associations between clinical conditions (*n* = 600) and MS diagnosis were assessed using Cox models adjusted for demographic variables, smoking, and MS‐PRS. Clinical conditions not appearing in at least five MS cases were excluded (*n* = 229), leaving 371 in the analysis. Figure 2 shows the statistical significance for all clinical conditions, while full results are presented in Table S4, including the HRs with 95% confidence intervals (CI), *p*‐values, relation with MS, and median time (in years) to MS diagnosis. Results showed that 192 clinical conditions were significantly associated with MS diagnosis (FDR < 0.05). As expected, typical MS onset symptoms (19%), such as demyelination, optic neuritis, and mobility issues, showed the strongest associations. However, several other potential prodromal conditions (56%), such as mental health disorders, cardiovascular diseases, genitourinary and gastrointestinal disorders, as well as other conditions with an unknown relationship with MS (21%), were also significantly associated with a higher MS diagnosis risk. MS risk factors (4%) included mononucleosis, viral CNS infection, tobacco abuse, head injury, and vitamin D deficiency, while pregnancy, measles, and viral warts were associated with a lower MS diagnosis risk.

**FIGURE 3:**
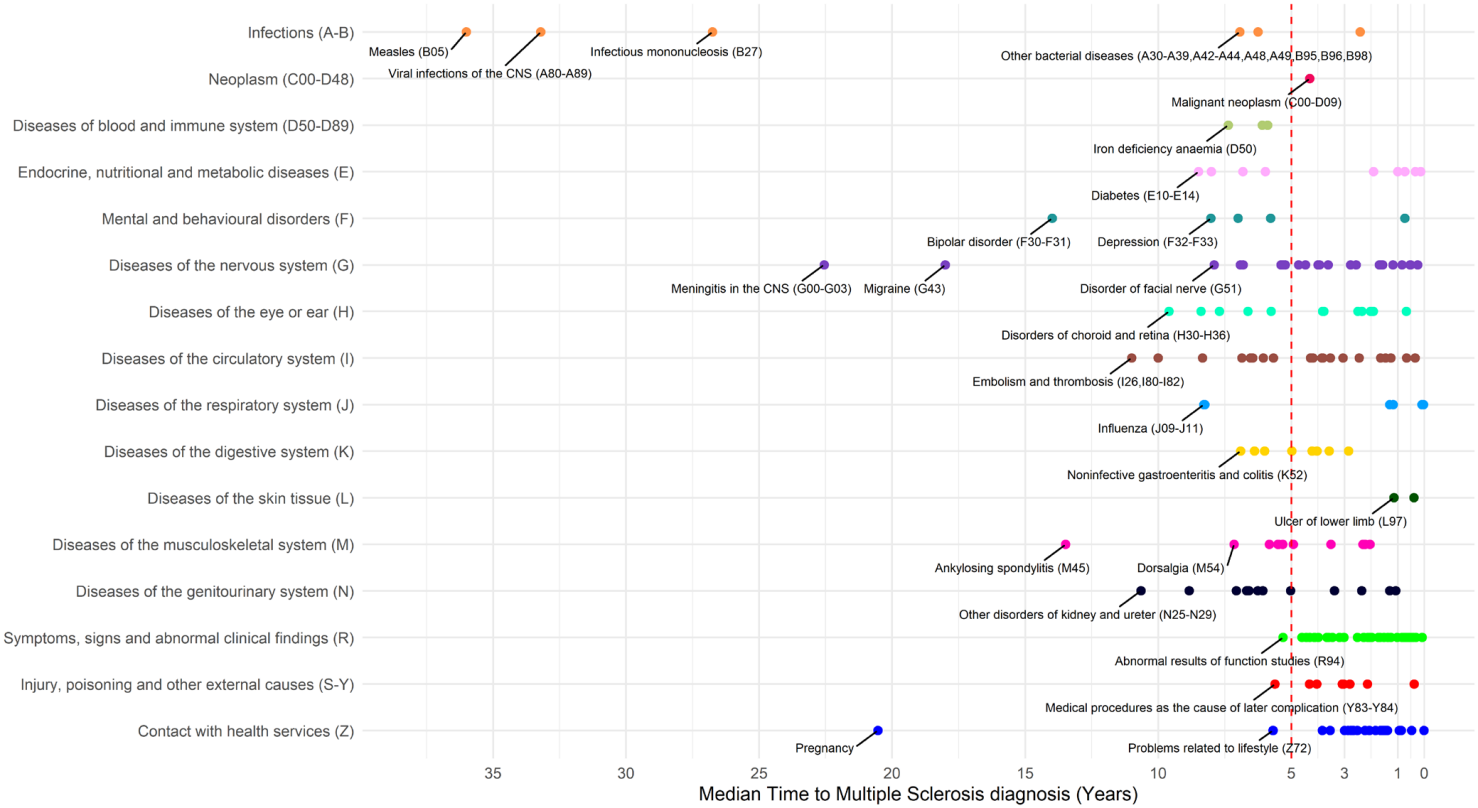
Timing of clinical conditions, grouped by different ICD‐10 code chapters, in relation to Multiple Sclerosis diagnosis. The timing was quantified as the median time, in years, between the clinical condition and Multiple Sclerosis diagnosis.

Table S5 summarizes the MS‐associated conditions by ICD‐10 code chapter and stratified by median time to MS diagnosis, and a graphical representation is also shown in Figure 3. Notably, 62 of the 192 significant conditions (32%) were diagnosed more than five years before MS, suggesting potential early indicators of MS risk. The earliest diagnoses, typically occurring in childhood and adolescence, were infections (e.g., measles, mononucleosis), while early conditions occurring in adulthood were mainly related to the genitourinary system (15%), nervous system (13%), cardiocirculatory system (13%), and musculoskeletal system (10%). Furthermore, these early signs also include mental health conditions like depression, anxiety, bipolar disorder, and tobacco abuse. In contrast, general symptoms and increased healthcare visits became more frequent in the five years immediately preceding MS diagnosis.

Excluding pregnancies, 60% of individuals with MS (*n* = 1485) had at least one MS‐associated condition before their diagnosis (median: 2, IQR: 1–4), with 1044 (42%) having more than one, totaling 8129 diagnoses. Absolute and relative frequencies for prior conditions in MS‐diagnosed individuals across ages and grouped by ICD‐10 code chapter are depicted in Figure S2 and provided in Table S6. Lastly, those with a history of MS‐associated conditions were diagnosed with MS at a later age (median: 50 years, IQR: 42–59) compared to those without prior conditions (median: 38 years, IQR: 30–44).

### 3.2 Evaluating the Predictive Accuracy of MS‐Associated Conditions

**FIGURE 4:**
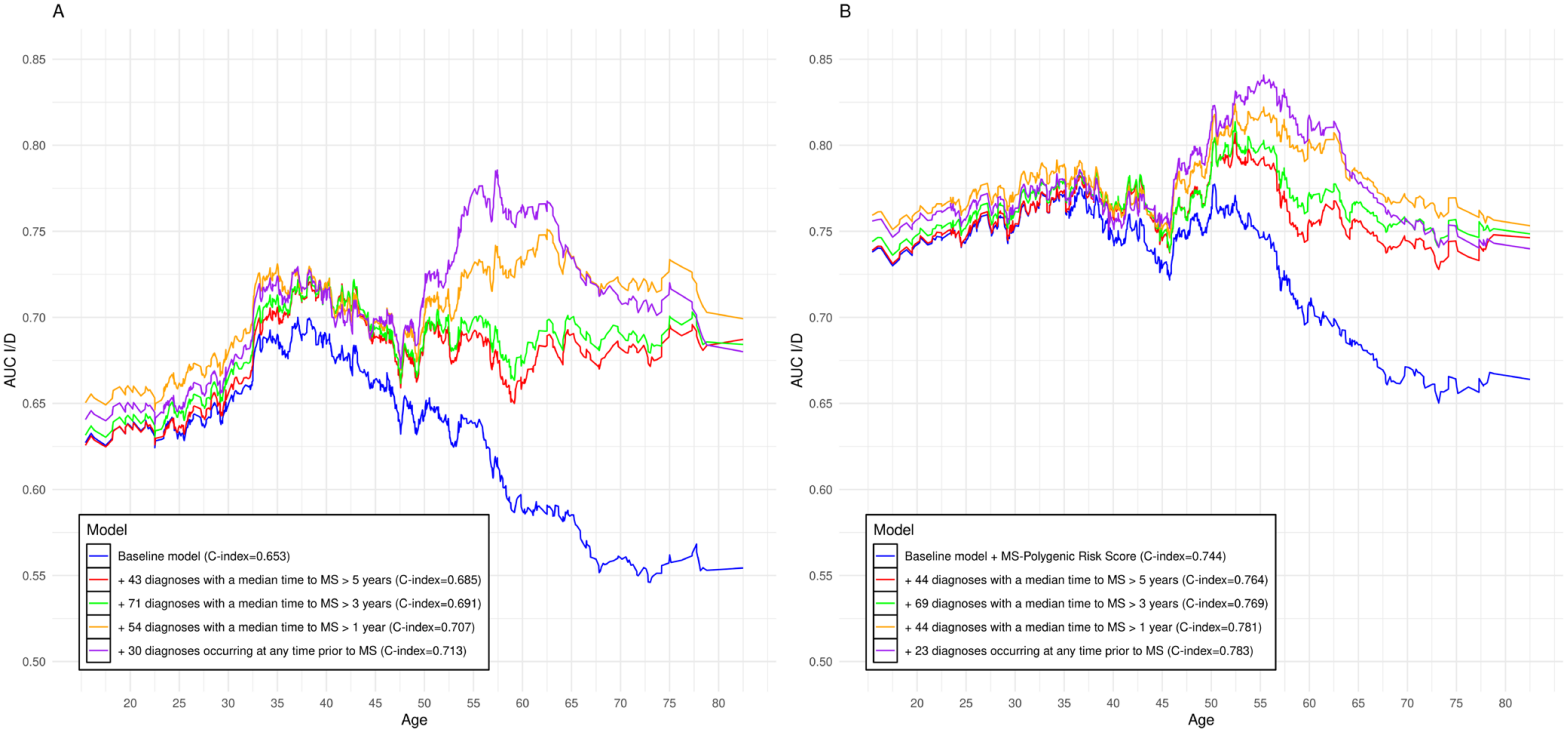
LASSO Cox models accuracy, in terms of C‐index and age‐dependent area under the curve (AUC I/D), due to the inclusion of clinical conditions significantly associated to Multiple Sclerosis diagnosis. Panel A refers to a baseline model including demographics and smoking, while panel B refers to a baseline model further including the genetic risk of Multiple Sclerosis. The predictive accuracy of the clinical conditions was evaluated based on their timing in relation to Multiple Sclerosis diagnosis, where the timing was quantified as the median time, in years, between the clinical condition and Multiple Sclerosis diagnosis.

**FIGURE 5:**
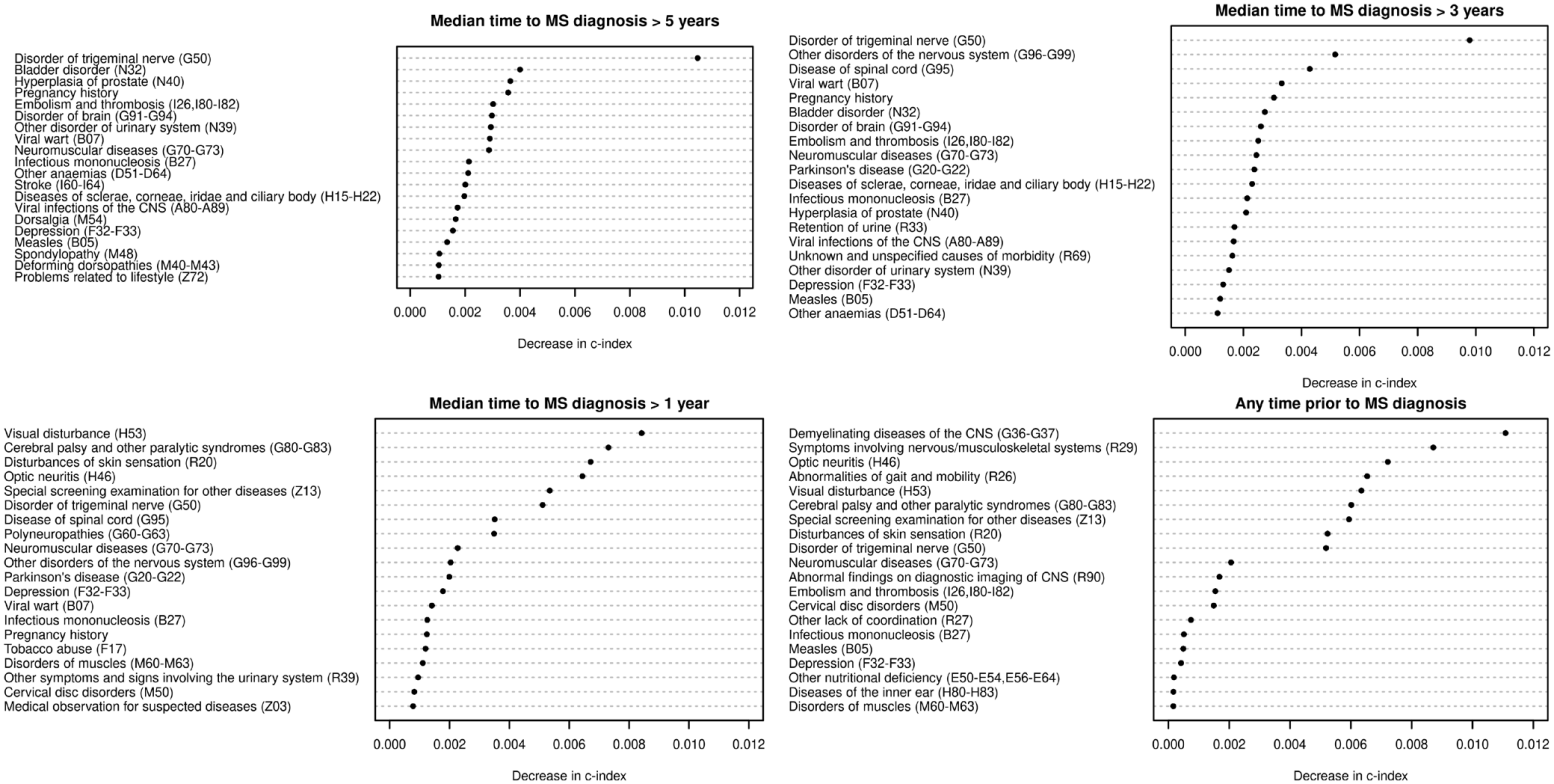
Top 20 predictive clinical conditions selected by LASSO Cox models, where clinical conditions were evaluated in stages based on their timing in relation to Multiple Sclerosis diagnosis, where the timing was quantified as the median time, in years, between the clinical condition and Multiple Sclerosis diagnosis.

We implemented predictive models to identify clinical conditions that could support earlier recognition of MS. The dataset was divided into a training/validation set (70%) and an external test set (30%). Using a LASSO Cox model, including demographics and smoking status (baseline model), we evaluated the improvement in predictive accuracy due to the inclusion of MS‐associated conditions. In a second time, we also incorporated the genetic MS risk (MS‐PRS) into the baseline model. Models’ performance in the test set, including C‐index values and age‐dependent AUC^I/D^ curves, are reported in Table S7 and Figure 4, respectively. The selected predictors and predictive value's ranking are reported in Tables S8–S11, while the top 20 predictors for each model are shown in Figure 5.

In the test dataset, the baseline model without MS‐PRS achieved a C‐index of 0.653. Adding 62 MS‐associated conditions that typically appeared more than five years before diagnosis led to the selection of 43 key conditions (Table S8), improving the accuracy to 0.685. The ranking based on the predictive value identified trigeminal neuralgia, an MS onset symptom, as the strongest predictive early condition, followed by several other prodromal conditions spanning multiple ICD‐10 chapters (Figure 5, top left), such as dorsalgia, bladder and prostate problems, depression, brain disorders, anemia, hypertension, pneumonia, diabetes, and problems related to lifestyle factors (e.g., addictions), early‐life infections (e.g., mononucleosis, viral CNS infection, measles), and pregnancy.

Adding conditions diagnosed three to five years and subsequently one to three years before MS increased the C‐index to, respectively, 0.691 (see Table S9 and Figure 5, top right), and 0.707 (see Table S10 and Figure 5, bottom left). As expected, conditions occurring closer to MS diagnosis included onset symptoms, such as spinal cord disease and optic neuritis. When all 192 MS‐associated conditions were included in the model, a final set of 30 conditions was selected, achieving a C‐index of 0.713 (see Table S11 and Figure 5, bottom right). Considering the timing of these conditions relative to MS diagnosis, 10 (33%) typically occurred more than three years before diagnosis, with 8 (27%) occurring over five years prior. In terms of their clinical relation with MS, 16 (53%) conditions were onset symptoms, 8 (27%) prodromal conditions, 3 (10%) risk factors, while 3 (10%) had an unknown relationship. These results further suggest the presence of predictive early and non‐specific indicators of MS. Among the eight predictive prodromal conditions, neuromuscular diseases, thromboembolism, and depression typically appeared more than 5 years before MS diagnosis, while the other prodromes typically occurred 1 to 3 years prior to MS diagnosis and included cervical disk disorders, muscle disorders, degenerative diseases of the basal ganglia, and lower limb ulcers. Additionally, nutritional deficiency was highlighted within 1 year of diagnosis. Lastly, the predictive risk factors included mononucleosis, viral CNS infection, and tobacco abuse, while the conditions with an unknown link to MS were measles, special screening examinations, and post‐surgical states.

When assessing predictive accuracy by age through the age‐dependent AUC^I/D^, the baseline model (without clinical history) peaked at ~68% accuracy between ages 30–40 but declined thereafter (Figure 4A, Table S7). In contrast, adding clinical history improved prediction from age 30 onward, reaching ~78% accuracy between ages 55–60. When genetic MS risk (MS‐PRS) was incorporated into the baseline model, the addition of clinical history improved the C‐index from 0.744 to 0.783. In particular, a substantial increase in predictive accuracy was observed after age 40, exceeding 80% between 50 to 65 years of age (Figure 4B, Table S7). Notably, after age 55, the model including clinical history, but without genetic risk, had a higher prediction accuracy compared to the model including the genetic risk alone.

### 3.3 Trajectories of MS‐Associated Conditions

Lastly, we examined the timing and sequence of the 192 MS‐associated conditions to understand their progression before MS diagnosis. Among the 1485 MS cases with at least one MS‐associated condition, the earliest recorded diagnoses were most often diseases of the nervous system (17%) and circulatory system (17%), with hypertension, depression, migraine, dorsalgia, and hypothyroidism being the most common initial conditions (see Figure S3A and Table S12 for full results). For the 1044 MS cases with at least two MS‐associated conditions, the last diagnosis before MS was most frequently a symptom or abnormal clinical finding (23%) or a nervous system disorder (15%). The most common final diagnoses included CNS demyelination, hypertension, urinary disorders, depression, and dorsalgia (see Figure S3B and Table S13 for full results). To better understand the progression of MS‐associated conditions, we analyzed 1144 diagnosis pairs occurring in at least 10 individuals, identifying 282 significant trajectories (see Table S14). The results revealed migraine as a central early condition, commonly followed by depression, hypertension, dorsalgia, and hypothyroidism. Two main clusters of diagnostic pathways emerged. The first cluster (Figure S4A) showed conditions such as hypertension, diabetes, dyslipidemia, urinary complications, and dorsalgia leading directly to MS diagnosis through a single step. Lipid metabolism disorders played a key role in linking hypertension to cerebrovascular diseases, hypotension, and renal failure. The second cluster (Figure S4B) was more complex, involving multiple intermediary conditions between early conditions and MS diagnosis. In this network, hypertension, dorsalgia, and hypothyroidism were mainly followed by disorders affecting the intestinal, musculoskeletal, cardiovascular, and visual systems. These conditions were then commonly followed by an increased healthcare utilization and the emergence of MS symptoms, such as fatigue, abnormal movements, dysphagia, sensory disturbances, and CNS demyelination. In contrast, depression was followed by CNS demyelination through lifestyle problems, anxiety, and neurological onset symptoms such as trigeminal neuralgia, mononeuropathies, and cerebral palsy. These findings suggest that specific early conditions, particularly migraine, hypertension, depression, and dorsalgia, may mark the beginning of prodromal trajectories leading to MS.

## 4 Discussion

This study used population‐cohort data from the UK Biobank study to provide novel insights into the clinical history preceding an MS diagnosis. By identifying 192 MS‐associated conditions and analyzing their temporal patterns, we found that MS diagnosis is often preceded by a distinct set of clinical manifestations, spanning multiple organ systems. Only a minority, about 20%, could be clearly identified as classical MS onset symptoms. Importantly, a substantial proportion of these conditions were observed well before the diagnosis of MS, as approximately one‐third occurred more than five years prior, and half were recorded at least three years before diagnosis. This temporal pattern reinforces the hypothesis that MS is preceded by a measurable prodromal phase during which subtle clinical changes may signal the emergence of MS.

The earliest conditions associated with MS were childhood and adolescent infections, which are more plausibly linked to the underlying pathogenesis of MS rather than representing early manifestations of the disease. Among these, mononucleosis and viral CNS infections were associated to a higher MS risk, consistently with the role of Epstein–Barr virus in MS development [29, 30, 31, 32, 33]. Instead, measles appeared to be associated with a lower risk, a finding that aligns partially with a previous study [34].

The associated clinical conditions appearing in adulthood are more likely to represent manifestations of a prodromal phase. Specifically, migraine emerged as a key early prodromal condition, supporting the results of a recent study [13], often preceding two predominant trajectories of disease progression. The first, a cardiovascular‐metabolic pathway, identified hypertension, diabetes, and dyslipidemia as early conditions subsequently followed by genitourinary disorders, a recognized MS prodromal manifestation. The second, a more heterogeneous pathway, identified cardiovascular (e.g., hypertension), endocrine (e.g., hypothyroidism), and neuropsychiatric (e.g., depression) disorders as early conditions, which then progressed to musculoskeletal, intestinal, visual, and neurological disorders before leading to classical MS onset symptoms such as motor dysfunction, sensory disturbances, and demyelination [35]. Together, these patterns suggest that the MS prodrome may begin several years before diagnosis, potentially reflecting subclinical demyelinating activity [36, 37].

The predictive value of these MS‐associated conditions was evaluated in models including demographics, smoking, and genetic MS risk (measured through an MS‐PRS). Genetic risk represented the strongest predictor in adulthood (AOMS), as the accuracy approached ~73% when including clinical history but not MS‐PRS, while it reached ~77% when including MS‐PRS but not clinical history. On the other hand, clinical history represented the strongest predictor in individuals over 50 years of age (LOMS), as the accuracy approached ~77% when including MS‐PRS but not clinical history, while it reached ~79% when including clinical history but not MS‐PRS. Nevertheless, accuracy further improved when both factors were included in the model, as the accuracy approached ~79% for AOMS cases and ~84% for LOMS cases. This suggests that younger MS cases, primarily driven by genetics [38, 39], have a more abrupt onset with fewer documented and potentially overlooked prodromal signs [38, 40, 41, 42], whereas older‐onset cases may follow a more gradual disease course with a longer prodromal phase. Both factors then provide distinct information in MS diagnosis prediction.

Interestingly, the improvement in prediction accuracy attributable to clinical history was already highlighted when limiting the analysis to early conditions typically occurring more than five years before diagnosis. Indeed, the identified disease trajectories showed that MS onset symptoms occurring closer to diagnosis were themselves predictable by earlier prodromal conditions. Notably, three early prodromal conditions occurring typically more than five years prior to MS diagnosis, i.e., neuromuscular diseases (such as myasthenia gravis), thromboembolism, and depression, were listed among the 30 best predictors of MS diagnosis. These conditions could highlight different aspects in the MS prodromal phase, such as autoimmunity (neuromuscular diseases [43]), vascular dysfunction (thromboembolism [44, 45]), and early signs of CNS demyelination (depression [46, 47]). Remarkably, trigeminal neuralgia [48, 49, 50], which emerged as the most predictive early‐onset MS symptom, was frequently preceded by depression and thromboembolism, as well as frequently followed by a diagnosis of CNS demyelination, representing a potential distinct clinical pathway toward MS diagnosis.

Other model‐selected prodromal conditions occurring one to three years prior to MS diagnosis included cervical disk and muscle disorders, degenerative diseases of the basal ganglia, and lower limb ulcers, alongside increased healthcare utilization. These conditions may reflect early manifestations of neurodegeneration, subtle neurological deficits, or non‐specific symptoms prompting contact with healthcare, leading to potential misdiagnoses before MS is recognized. Their identification as potential warning signs could provide critical opportunities for earlier recognition and intervention. Lastly, the most predictive early conditions included measles and known risk factors, i.e., mononucleosis, viral CNS infections, and tobacco abuse (which may relate to both cigarette smoking and neuropsychiatric disorders [51, 52, 53]).

These findings, obtained on a large sample size with recorded lifetime clinical histories, may have important clinical implications. First, they reinforce the hypothesis that MS diagnosis is preceded by an identifiable prodromal phase. Second, the integration of clinical history into predictive tools for MS diagnosis in clinical practice could enhance earlier screening and intervention in high‐risk individuals. Third, the high predictive accuracy reached in individuals aged > 50 years suggests that clinical history is particularly valuable for improving the identification of LOMS cases, the incidence of which is recently increasing and which are often misdiagnosed due to atypical presentations [20, 21, 22].

Despite the interesting findings, several limitations must be acknowledged. Reliance on ICD‐10‐coded diagnoses limit the generalizability of our results to conditions severe enough to require medical attention, as symptoms and signs that do not reach clinical attention were not systematically recorded. Moreover, diagnoses retrospectively obtained from healthcare records and self‐reports at the time of recruitment may be underreported, especially for those dating back to early life (e.g., childhood infections). On the other hand, this does not seem the case for MS, as the alignment of MS prevalence in the UK Biobank with national estimates suggests accurate case identification [54]. Lastly, the predominant European ancestry of the UK Biobank limits generalizability to other populations. Despite these limitations, our findings emphasize the value of early and prodromal conditions in improving MS detection. Future research should validate these results in independent cohorts, and explore how integrating subtle signs and symptoms, lifestyle factors, and biomarkers could further refine and enhance the accuracy for an MS prediction tool to be implemented and tested in the clinical practice.

## Author Contributions

Conceptualization: Andrea Nova, Teresa Fazia, Giovanni Di Caprio, Roberto Bergamaschi. Data curation: Andrea Nova. Formal analysis: Andrea Nova, Teresa Fazia. Methodology: Andrea Nova, Teresa Fazia. Software: Andrea Nova. Visualization: Andrea Nova. Writing – original draft: Andrea Nova. Writing – review and editing: all the authors. Supervision: Roberto Bergamaschi.

## Conflicts of Interest

The authors declare no conflicts of interest.

## Supporting information


Data S1.

Data S2.

Data S3.

Figure S1.

Figure S2.

Figure S3.

Figure S4.

Text S1.

## Data Availability

Researchers may obtain access to the data used in this study upon reasonable request to the UK Biobank study team (https://www.ukbiobank.ac.uk/).
