# Supplementary material for: Timing and Predictive Value of Clinical Conditions Preceding Multiple Sclerosis in the UK Biobank
Source: Ann Clin Transl Neurol. 2025 Jun 26;12(10):1952–61. doi: 10.1002/acn3.70119 (PMC12516241; doi:10.1002/acn3.70119)
Supplement: Supplementary file 1 — Data S1. [file ACN3-12-1952-s007.docx]

#########################

#R code template for the statistical analysis described in "Timing and predictive value of clinical conditions preceding Multiple Sclerosis in the UK Biobank"

#Code written by Andrea Nova (University of Pavia).

#For any question, you can contact me at this mail: andrea.nova01@universitadipavia.it

#########################

# You can copy and paste this code into your R environment for improved clarity.

#This R script represents a simplified template of our analyses with all relevant instructions to replicate the analysis.

#Furthermore, it can be adapted for all outcomes and covariates of interest.

##################################

#Data Management

#Data could be obtained only with a project application to the UK Biobank team.

#Load the library

library(survival)

#Code to obtain a dataset with clinical conditions and smoking initiation as time-varying variables.

#Data requires the following elements:

# ID: subject's ID.

# timetoMS: observation time from start to follow-up to MS diagnosis or censoring.

# MS: Binary indicator of the Multiple Sclerosis diagnosis (0/1).

# AgeStartSmoking: Age when subject started to smoke. If the subject is not a smoker the value is NA.

# AgeCondition1: Age when subject has been diagnosed with a specific clinical condition. If the subject had not been diagnosed with that condition the value is NA.

# AgeCondition2, AgeCondition2, etc...: same as AgeCondition1 but for other clinical conditions.

data2<-tmerge(data,data, id=ID, MS_2=event(timetoMS,MS))

temp<-tmerge(data2,data, id=ID,

Smoke=tdc(AgeStartSmoking),

Condition1=tdc(AgeCondition1),

Condition2=tdc(AgeCondition2),

Condition3=tdc(AgeCondition3),.....all other conditions of interest)

#The dataset "temp" now contains repeated rows for each subject based on the time-varying variables.

#tstart and tstop represent the interval time, while covariates are equal to 1 if they are present at the start of the interval.

# Outcome (MS_2) is equal to 1 if the subject has been diagnosed at the end of the interval.

#Example

#ID tstart tstop MS_2 Smoke Condition1 Condition2 Condition3

#1 0 16 0 0 0 0 0

#1 16 41 0 1 1 0 0

#1 41 71 0 1 1 0 0

#2 0 25 1 0 0 0 0

#3 0 24 0 0 0 0 0

#3 24 44 0 0 0 1 0

#3 44 60 0 0 0 1 1

####################################################################################################################################

####################################################################################################################################

#Analysis 1

#Univariate Cox model to evaluate the association between a clinical condition and MS, adjusting for other confounders.

model <- coxph(Surv(tstart,tstop,MS_2) ~ Condition1+Sex+Year_of_birth+Smoke+Ethnicity+CountryBirth+MSPRS+cluster(ID), data = temp)

summary(model)

####################################################################################################################################

#Analysis 2

#Predictive analysis through a LASSO Cox model with 5-folds CV

#Load relevant libraries

library(caret)

library(glmnet)

#For reproducibility

set.seed(123)

#Step 1: Split the dataset into 70% training/validation and 30% test

train_indices <- sample(1:nrow(data), size = 0.7 * nrow(data)) #this vector contains the row numbers of the individuals in the training/validation set.

eid<-data$ID[train_indices]

train_data <- temp[which(temp$ID %in% eid), ] #Training/validation dataset

test_data <- temp[-which(temp$ID %in% eid), ] #Test dataset

#Step 2: Create K=5 folds with similar number of MS cases within each fold

data2<-data[which(data$ID %in% eid),]

folds <- createFolds(y = data2$MS, k = 5, list = FALSE)

data2$folds<-folds

train_data<-merge(train_data,data2[,c("eid","folds")], by="ID", all.x=T)

#Step 3: LASSO Cox model implementation with 5-folds Cross Validation

#Use multiple cores for higher computational efficiency

library(doMC)

unregister_dopar <- function() {

env <- foreach:::.foreachGlobals

rm(list=ls(name=env), pos=env)

}

unregister_dopar()

registerDoMC(cores = 5)

#Create an object retaining only the predictors of interest (outcome and ID are excluded!)

train_data2<-train_data[,Select the predictor of interest]

#Example

#train_data2<-train_data[,c("Condition1","Condition2","Condition3","Sex","Smoke")]

#Create a sparse matrix with the predictors of interest to improve computational efficiency

x_sparse <- as(as.matrix(train_data2),"sparseMatrix")

#Create the outcome based on the time to MS diagnosis.

yss <- Surv(train_data$tstart, train_data$tstop, train_data$MS_2)

#5-folds CV Cox model

fit <- cv.glmnet(x_sparse, yss, family = "cox", nfolds=5, parallel=TRUE, trace.it = TRUE, maxit=10^8, type.measure ="deviance",foldid = train_data$folds )

#Calculating accuracy through the C-index in the test set

test_data2<-test_data[,Select the predictors of interest as in train_data2]

x_sparse2 <- as(as.matrix(test_data2),"sparseMatrix")

yss2 <- Surv(test_data$tstart, test_data$tstop, test_data$MS_2)

preds <- predict(fit, newx = x_sparse2,s = "lambda.min",type = "link")

Cindex(preds, yss2) #C-index in the test set.

####################################################################################################################################

#Permutations to obtain variable importance of the selected LASSO-predictors

#Step 1: Save the C-index calculated in the test set into an object "bas"

bas<-Cindex(preds, yss2)

#Step 1: Select the LASSO-selected predictors from the 5-folds CV LASSO Cox model

lasso_coefs <- coef(fit, s = "lambda.min")

selected_features <- as.data.frame(as.matrix(lasso_coefs))

selected_features <- selected_features[selected_features[,1] != 0, , drop = FALSE] # Retain non-zero coefficients

print(selected_features) #LASSO-selected predictors

#Step 2: Create an index variable based on the columns' number in the dataset "test_data" corresponding to the LASSO-selected predictors. This is required to implement the following function permute_importance_cox

SIG2<-rownames(selected_features)

index<-which(names(test_data) %in% SIG2) #This simply indicates the column numbers in "test_data" corresponding to the LASSO-selected predictors

#Step 3: Save the following function which creates 500 permutations for each LASSO-selected predictor.

permute_importance_cox <- function(model, x, baseline_performance) {

importance_list <- mclapply(1:500, function(i) {

importance <- numeric(ncol(x))

for (j in index) {

x_permuted <- x

x_permuted[, j] <- sample(x[, j]) # Permute the j-th variable

# Predict with permuted data

permuted_preds <- predict(model, newx = x_permuted, s = "lambda.min", type = "link")

permuted_cindex <- Cindex(permuted_preds, yss2)

# Calculate importance as the change in C-index

importance[j] <- baseline_performance - permuted_cindex

}

return(importance)

}, mc.cores = 10) # Use available cores

# Combine results

importance_matrix <- do.call(cbind, importance_list)

return(importance_matrix)

}

#Step 4: Run the "permute_importance_cox" function to obtain the Variable Importance for each LASSO-selected predictor over 500 permutations.

variable_importance <- permute_importance_cox(fit, as.matrix(test_data2), bas)

#Step 5: Take the average over all the 500 permutations.

variable_importance<-rowMeans(variable_importance)

#Step 6: For improved clarity, this step creates an object "VIMP" with decreasing variable importance so that the top predictors are the most important.

variable_importance<-variable_importance[which(variable_importance!=0)]

VIMP<-as.data.frame(cbind(variable_importance,SIG2))

VIMP$variable_importance<-as.numeric(VIMP$variable_importance)

VIMP<-VIMP[order(VIMP$variable_importance, decreasing=F),]

#Step 7: Draw a plot with the top 20 predictors.

dotchart(VIMP$variable_importance[(nrow(VIMP)-19):nrow(VIMP)], VIMP$SIG2[(nrow(VIMP)-19):nrow(VIMP)],

pch=16,xlab="Decrease in c-index",cex=0.5)

#Step 8: Function to estimate the Age-dependent AUC.

library(risksetROC)

currLambdaOS<-0.2 #Lambda parameter fixed to 0.2. Lower values increase potential overfitting.

test_data$preds<-preds #predicted survival time (preds) in the test dataset has been created in the steps above

mmmTV <- MeanRank(survival.time= test_data$tstop, survival.status= test_data$MS_2, marker=test_data$preds,

start= test_data$tstart)

nnn <- nne(x= mmmTV$time, y= mmmTV$mean.rank, lambda=currLambdaOS, nControls=mmmTV$nControls) #nnn contains AUC as a function of age

#Step 8: Draw a plot of Age-dependent AUC.

plot(nnn$x, nnn$nne, type="l", col="blue", ylim=c(0.5,0.9), xlab="Age", ylab="AUC I/D")

####################################################################################################################################

#Trajectory Analysis

#Step 1: Only MS cases are retained

dataMS<-data[which(data$MS==1),]

A1<-SignificantConditions #SignificantConditions is simply a vector with the name of the significant MS-associated conditions based on Analysis 1

#Step 2: Calculate all possible trajectories D1 -> D2 based on the age at D1 and D2 diagnosis.

"%nin%"<-Negate("%in%") #Function needed to find elements in the first vector not included in the second vector (needed in the following function)

TRAJ<-NULL #Initialize an object to store all relevant information for the trajectories D1 -> D2

for(i in 1:length(A1)){

A2 <- A1[which(A1 %nin% A1[i])]

AgeA1<-paste0("Age",A1[i])

for (j in 1:length(A2)) {

AgeA2<-paste0("Age",A2[j])

D11<-length(which(dataMS[[A1[i]]]==1))

D22<-length(which(dataMS[[A2[j]]]==1))

D12<-length(which(dataMS[[AgeA2]] > dataMS[[AgeA1]] & dataMS[[A1[i]]]==1 & dataMS[[A2[j]]]==1))

D21<-length(which(dataMS[[AgeA1]] > dataMS[[AgeA2]] & dataMS[[A1[i]]]==1 & dataMS[[A2[j]]]==1))

DTOT<-D12+D21

TRAJ<-rbind(TRAJ,c(A1[i],A2[j],D11,D22,D12,D21,DTOT))

}

}

TRAJ<-as.data.frame(TRAJ)

#Recode nummeric variables.

TRAJ$V3<-as.numeric(TRAJ$V3)

TRAJ$V4<-as.numeric(TRAJ$V4)

TRAJ$V5<-as.numeric(TRAJ$V5)

TRAJ$V6<-as.numeric(TRAJ$V6)

TRAJ$V7<-as.numeric(TRAJ$V7)

#Change column names to improve clarity.

names(TRAJ)[1]<-"D1" #Condition 1 (D1)

names(TRAJ)[2]<-"D2" #Condition 2 (D2)

names(TRAJ)[3]<-"ND1" #Number of subjects with Condition 1 (D1)

names(TRAJ)[4]<-"ND2" #Number of subjects with Condition 2 (D2)

names(TRAJ)[5]<-"D12" #Number of subjects with Condition 1 (D1) before Condition 2 (D2)

names(TRAJ)[6]<-"D21" #Number of subjects with Condition 2 (D2) before Condition 2 (D1)

names(TRAJ)[7]<-"DTOT" #Number of subjects with both Condition 1 (D1) and Condition 2 (D2)

#Step 3: Keep only trajectories D1->D2.

TRAJ<-TRAJ[which(TRAJ$D12>=TRAJ$D21),]

#Step 4: Keep only trajectories occurring in at least 10 subjects.

TRAJ<-TRAJ[which(TRAJ$DTOT>=10),]

#Step 5: Fisher Test to obtain a p-value for the statistical significance of the trajectories D1->D2

TRAJ$Fisher_pvalue<-NA

for(i in 1:nrow(TRAJ)){

TRAJ$Fisher_pvalue[i]<-fisher.test(matrix(

c(TRAJ$D12[i], TRAJ$DTOT[i] - TRAJ$D12[i],

TRAJ$D21[i], TRAJ$DTOT[i] - TRAJ$D21[i]),

nrow = 2, byrow = TRUE

), alternative = "greater")$p.value

}

#Step 6: Calculate Odds Ratio for the trajectory D1->D2 using a conditional logistic model based on a nested case-control design.

#Initialize objects to store Odds Ratio, Standard Error and p-value.

TRAJ$OR12<-NA

TRAJ$pval12<-NA

TRAJ$se12<-NA

#Cycle to implement the analysis for each trajectory.

library(dplyr)

library(tidyverse)

for(i in 1:nrow(TRAJ)){

D1<-TRAJ$D1[i]

D2<-TRAJ$D2[i]

AgeD1<-paste0("Age",D1)

AgeD2<-paste0("Age",D2)

#Select cases as the individuals diagnosed with D2 after D1.

dataMS1<-dataMS[which(dataMS[[D2]]==1),]

cases <- dataMS1 %>%

mutate(case = 1)

cases[which(cases[[AgeD1]]>=cases[[AgeD2]] & cases[[D2]]==1 & cases[[D1]]==1),D1]<-0

cases$strat<-1:nrow(cases)

#Select controls as the individuals not diagnosed with D2 or diagnosed with D2 before D1.

controls <- dataMS[which(dataMS[[D2]]==0),]

controls[which(controls[[AgeD1]]>=controls[[AgeD2]] & controls[[D2]]==1 & controls[[D1]]==1),D1]<-0

#Match cases to controls based on the maximum number of controls at the age at D2 diagnosis.

s<-floor(nrow(dataMS)/nrow(cases))-1 #Maximum number of matched controls.

controls<- controls %>%

sample_n(size = nrow(cases)*s, replace = F) %>% # Randomly match cases to controls

mutate(case = 0)

controls$strat<-rep(1:nrow(cases),s)

controls[[AgeD2]]<-NULL

controls<-merge(controls,cases[,c("strat",AgeD2)], by="strat",all.x=T)

controls[which(controls[[AgeD1]]>=controls[[AgeD2]] & controls[[D1]]==1),D1]<-0

#Combine cases and controls

nested_case_control <- bind_rows(cases, controls)

#Fit Conditional Logistic model adjusted for confounders as in Analysis 1.

expr_string <- paste0("clogit(case ~ ",D1," + Sex+Smoker+MSPRS+Year_of_birth+Ethnicity+CountryBirth +strata(strat), data = nested_case_control)")

clogit_model <-eval(parse(text = expr_string))

clogit_model <-summary(clogit_model)

TRAJ$OR12[i]<-clogit_model$coefficients[1,2] #Odds Ratio

TRAJ$se12[i]<-clogit_model$coefficients[1,3] #Standard Error

TRAJ$pval12[i]<-clogit_model$coefficients[1,5] #p-value

print(i)

}

#The object TRAJ now contains all relevant information for trajectories D1->D2.

#D1: Condition 1 (D1) name

#D2: Condition 2 (D2) name

#ND1: Number of subjects with Condition 1 (D1)

#ND2: Number of subjects with Condition 2 (D2)

#D12: Number of subjects with Condition 1 (D1) before Condition 2 (D2)

#D21: Number of subjects with Condition 2 (D2) before Condition 1 (D1)

#DTOT: Number of subjects with both Condition 1 (D1) and Condition 2 (D2)

#Fisher_pvalue: p-value for the trajectory D1->D2

#OR12: Odds Ratio for the strength of the association for the trajectory D1->D2

#se12: Standard Error for the estimated Odds Ratio

#pval12: p-value based on the estimated Odds Ratio for the trajectory D1->D2.

#######################################################################################

#Multiple testing adjustment. All p-values were adjusted based on the False Discovery Rate control at 0.05.

p.adjust(pvalues, method="BH)
