# Supplementary material for: Timing and Predictive Value of Clinical Conditions Preceding Multiple Sclerosis in the UK Biobank
Source: Ann Clin Transl Neurol. 2025 Jun 26;12(10):1952–61. doi: 10.1002/acn3.70119 (PMC12516241; doi:10.1002/acn3.70119)
Supplement: Supplementary file 2 — Data S2. [file ACN3-12-1952-s001.docx]

**Supplemental Material for “Timing and predictive value of clinical conditions preceding Multiple Sclerosis in the UK Biobank”**

**Methods**

**Evaluating the predictive accuracy of MS-associated conditions**

To identify the most predictive MS-associated conditions and assess their contribution to MS prediction, we implemented a two-step modeling approach. The dataset was split into a training/validation set (70%) and an external test set (30%). A baseline model was first developed using sex, year of birth, ethnicity, place of birth, and smoking history as predictors. We applied LASSO Cox regression with five-fold cross-validation (CV) in the training set, selecting the optimal lambda parameter that minimized the partial likelihood deviance function. The predictive accuracy of this model was evaluated in the test set using Harrell’s C-index (C_1_), which estimates the probability that an individual diagnosed with MS has a higher predicted MS risk than one diagnosed later^1^. The R package *glmnet* and its *cv.glmnet* function were used to implement LASSO Cox regression.

In the second step, we repeated the modeling process by sequentially adding MS-associated conditions as time-varying covariates. To better characterize the predictive value of the conditions based on their timing in relation to MS diagnosis, we included the conditions among the predictors in stages based on their median time to MS diagnosis: i) more than 5 years before MS, ii) more than 3 years before MS, iii) more than 1 year before MS, iv) any time before MS. In each scenario, a new C-index (C_2_) was calculated in the test dataset, and the improvement in predictive accuracy was determined by the difference (C_2_ – C_1_). To rank the conditions based on their predictive value, we assessed the importance of each selected predictor using permutation testing^2^. Each variable, i.e., the diagnosis of the clinical condition, was randomly permuted 500 times, and the C-index (C_perm_) was recalculated on the test dataset. The average difference between C_perm_ and C_2_ quantified the decrease in accuracy, providing an estimate of the relative variable’s importance (VI) in predicting MS, i.e., a higher VI implies a higher ranking in predictive value. Stages i), ii), and iii) allowed us to evaluate predictive improvement after the addition of clinical condition occurring in different time windows prior to MS diagnosis, while step iv) represented the final model which considered conditions occurring at any time.

Lastly, the entire two-step modeling process was repeated with MS-PRS included in the baseline model to assess whether clinical history improved prediction beyond genetic risk alone. For each model, we calculated the time-dependent Incident Cases / Dynamic Controls Area Under the Curve (AUC^I/D^(t)) to evaluate predictive accuracy across different ages. AUC^I/D^(t) represents the probability that an individual diagnosed with MS at age t has a higher predicted MS risk score than an individual of the same age who is MS-free at that time^3^. We estimated AUC^I/D^(t) using a nonparametric rank-based approach with a nearest-neighbor kernel and a smoothing parameter λ_n_ = 0.20. The R package *risksetROC* R package and it function *nne* were used to calculate AUC^I/D^(t).

**References:**

1. Longato E, Vettoretti M, Di Camillo B. A practical perspective on the concordance index for the evaluation and selection of prognostic time-to-event models. J. Biomed. Inform. 2020;108

2. Altmann A, Toloşi L, Sander O, Lengauer T. Permutation importance: A corrected feature importance measure. Bioinformatics 2010;26(10)

3. Bansal A, Heagerty PJ. A Tutorial on Evaluating the Time-Varying Discrimination Accuracy of Survival Models Used in Dynamic Decision Making. Med. Decis. Mak. 2018;38(8)
