# Supplementary figures and images for: Timing and Predictive Value of Clinical Conditions Preceding Multiple Sclerosis in the UK Biobank
Source: Ann Clin Transl Neurol. 2025 Jun 26;12(10):1952–61. doi: 10.1002/acn3.70119 (PMC12516241; doi:10.1002/acn3.70119)

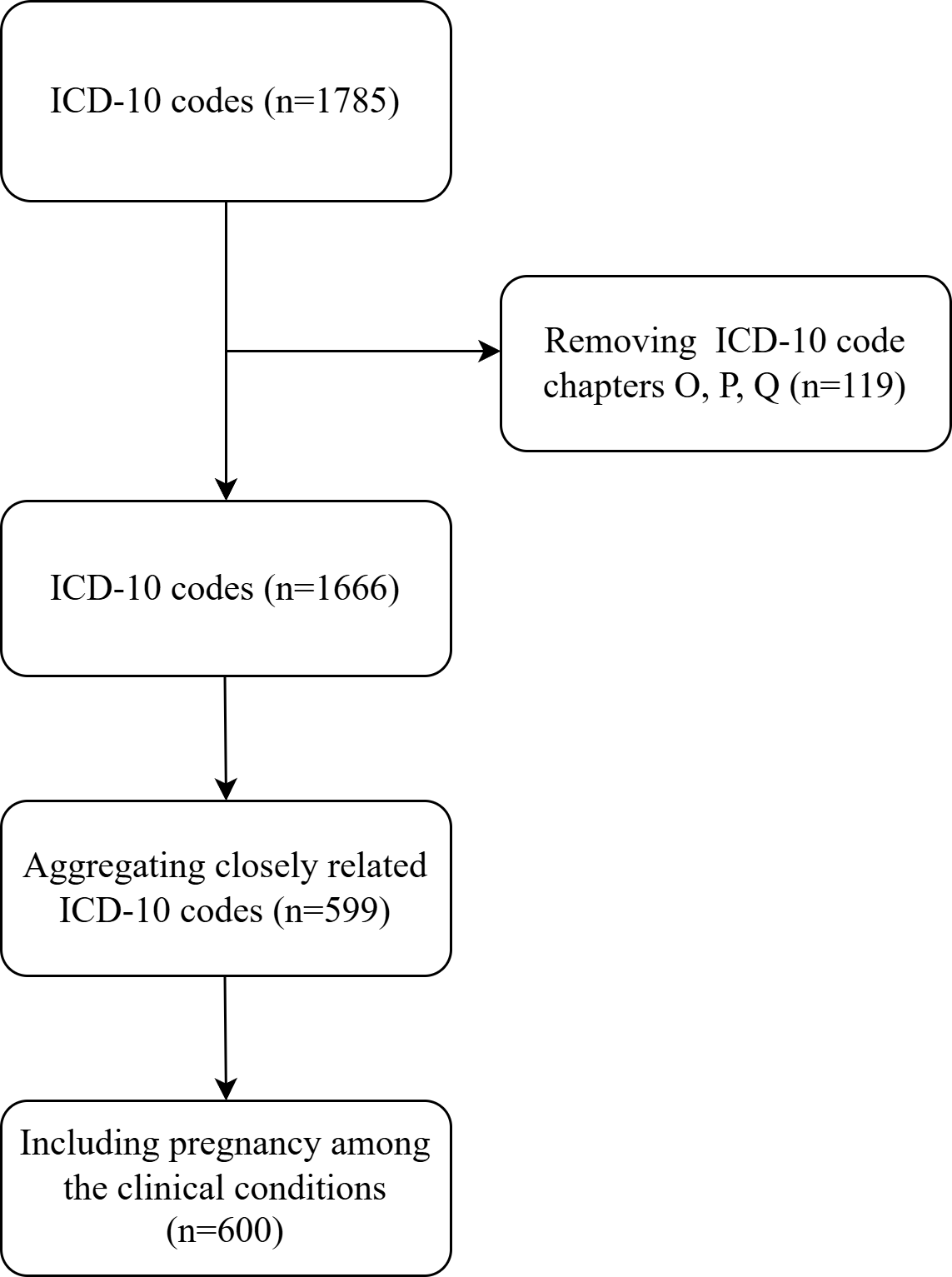

Supplement: Supplementary file 4 — Figure S1. [file ACN3-12-1952-s004.png]

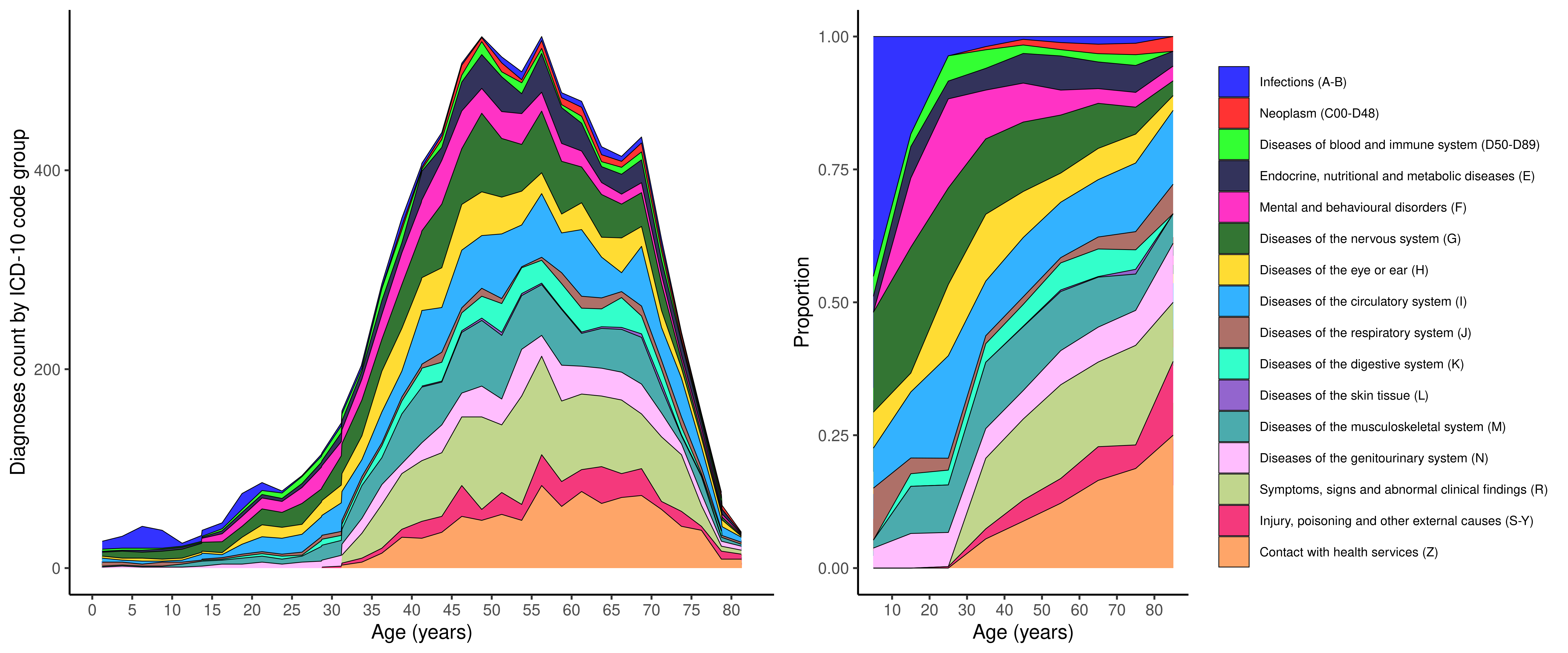

Supplement: Supplementary file 5 — Figure S2. [file ACN3-12-1952-s005.tif]

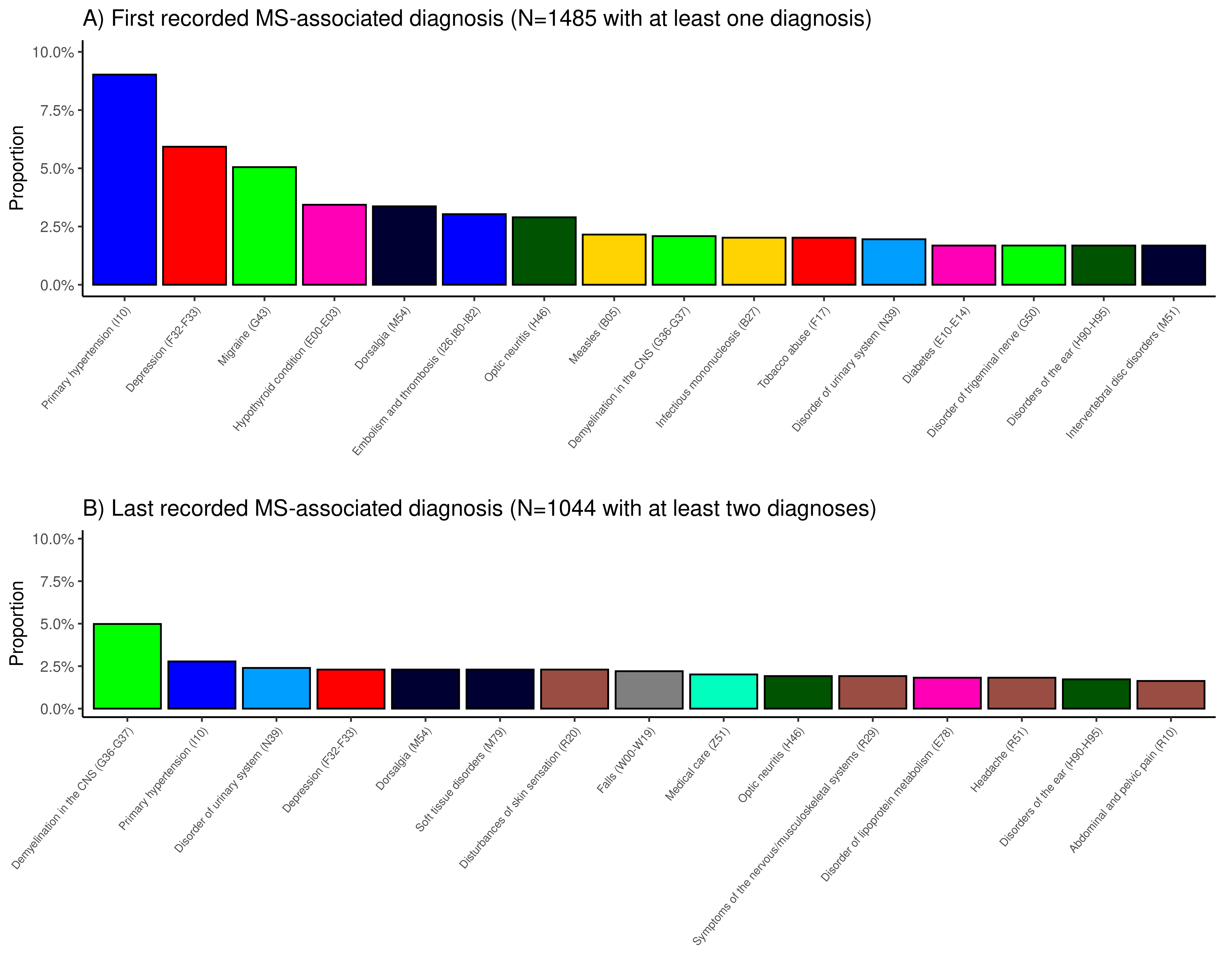

Supplement: Supplementary file 6 — Figure S3. [file ACN3-12-1952-s002.tif]

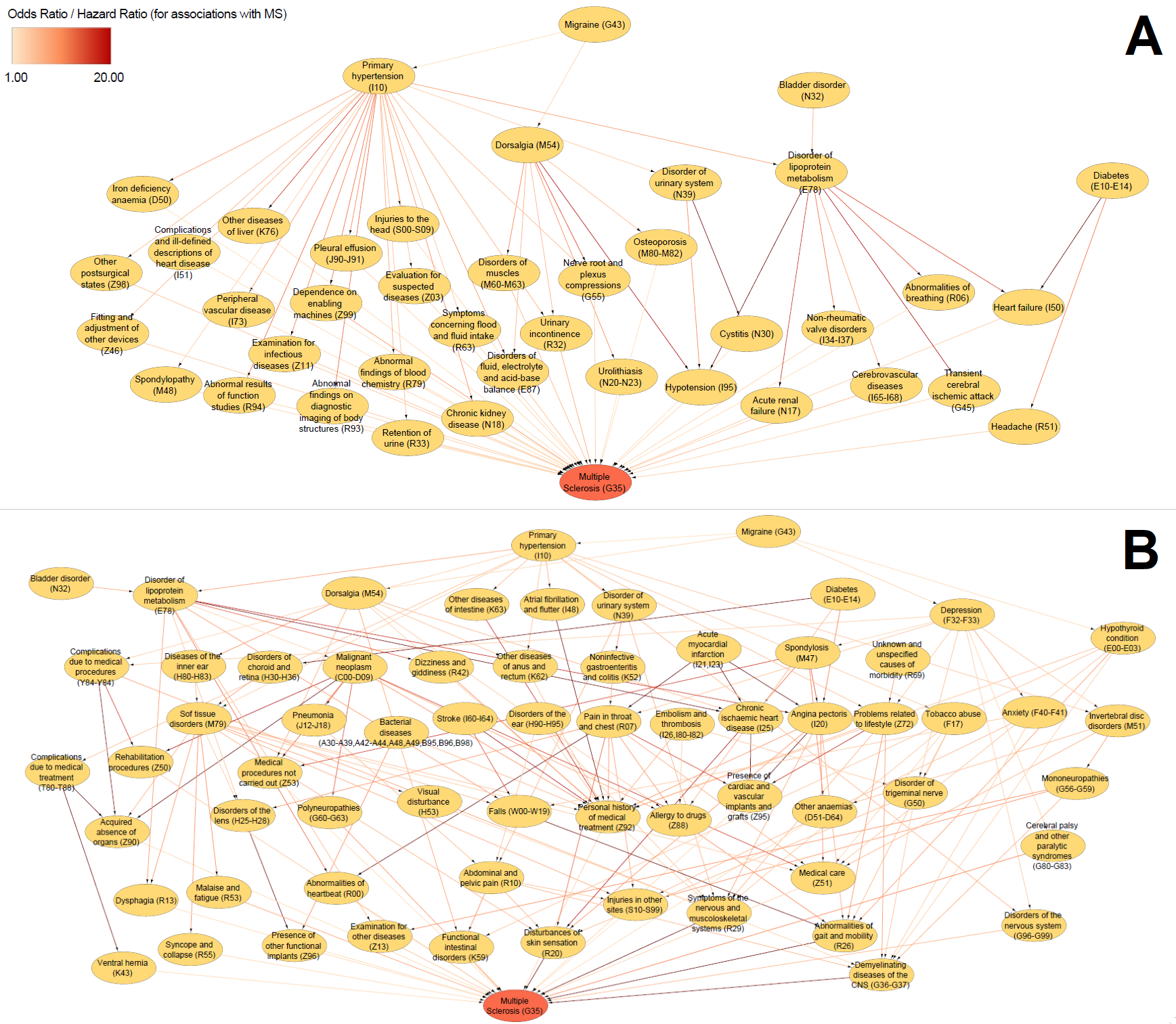

Supplement: Supplementary file 7 — Figure S4. [file ACN3-12-1952-s003.tiff]
