# Supplementary material for: Timing and Predictive Value of Clinical Conditions Preceding Multiple Sclerosis in the UK Biobank
Source: Ann Clin Transl Neurol. 2025 Jun 26;12(10):1952–61. doi: 10.1002/acn3.70119 (PMC12516241; doi:10.1002/acn3.70119)
Supplement: Supplementary file 8 — Text S1. [file ACN3-12-1952-s008.docx]

Supplemental Figure 1: Flowchart describing the filtering process to obtain, from ICD-10 codes, the clinical conditions used in the analysis.

Supplemental Figure 2: Absolute and cumulative relative frequencies for clinical conditions preceding Multiple Sclerosis diagnosis across ages and grouped by ICD-10 code chapter.

Supplemental Figure 3: Relative frequencies for earliest and last recorded clinical condition in Multiple Sclerosis diagnosed individuals.

Supplemental Figure 4: Clusters of clinical conditions trajectories leading to Multiple Sclerosis diagnosis. A) The first cluster shows a cardiovascular-metabolic pathway. B) The second cluster was more complex, involving cardiovascular, endocrine, and neuropsychiatric disorders. As shown in the legend, the color of the arrow linking clinical conditions denotes the strength of the association.
